# Effect of betanin synthesis on photosynthesis and tyrosine metabolism in transgenic carrot

**DOI:** 10.1186/s12870-023-04383-9

**Published:** 2023-08-24

**Authors:** Bo Wang, Ya-Hui Wang, Yuan-Jie Deng, Quan-Hong Yao, Ai-Sheng Xiong

**Affiliations:** 1 State Key Laboratory of Crop Genetics & Germplasm Enhancement and Utilization, College of Horticulture, Nanjing Agricultural University, Nanjing, 210095 China; 2 Shanghai Key Laboratory of Agricultural Genetics and Breeding, Biotechnology Research Institute, Shanghai Academy of Agricultural Science, Shanghai, 201106 China

**Keywords:** Genetically modified carrot, Betanin, Unintended effects, Photosynthesis, Transcriptome, Metabolomics

## Abstract

**Background:**

Betalain is a natural pigment with important nutritional value and broad application prospects. Previously, we produced betanin biosynthesis transgenic carrots *via* expressing optimized genes *CYP76AD1S*, *cDOPA5GTS* and *DODA1S*. Betanin can accumulate throughout the whole transgenic carrots. But the effects of betanin accumulation on the metabolism of transgenic plants and whether it produces unexpected effects are still unclear.

**Results:**

The accumulation of betanin in leaves can significantly improve its antioxidant capacity and induce a decrease of chlorophyll content. Transcriptome and metabolomics analysis showed that 14.0% of genes and 33.1% of metabolites were significantly different, and metabolic pathways related to photosynthesis and tyrosine metabolism were markedly altered. Combined analysis showed that phenylpropane biosynthesis pathway significantly enriched the differentially expressed genes and significantly altered metabolites.

**Conclusions:**

Results showed that the metabolic status was significantly altered between transgenic and non-transgenic carrots, especially the photosynthesis and tyrosine metabolism. The extra consumption of tyrosine and accumulation of betanin might be the leading causes.

**Supplementary Information:**

The online version contains supplementary material available at 10.1186/s12870-023-04383-9.

## Background

Carrot (*Daucus carota* L.) is a biennial root vegetable belonging to the Apiaceae family and originated from Western Asia (Afghanistan) [1]. Fleshy root of carrot is its main edible part, which is rich in sugars, fats, vitamins, anthocyanins, calcium, iron, and other trace elements. The cultivated carrots are divided into eastern and western carrots according to their pigmentation synthesized in fleshy roots. Differences in the composition of carotenoids and anthocyanins in fleshy roots give rise to a variety of color types, including orange, yellow, red, purple, and white [2, 3]. Carrot, used as a fresh or processing vegetable, is one of the world’s top vegetable crops and has been cultivated worldwide due to its wide adaptability and strong resistance [4]. In addition, the annual cultivated area and total production of carrot during 2020 were 1.13 million ha and 41 million tons (carrots and turnips) [5]. The yields of carrots are much higher than those of staple food crops, such as maize, rice, and wheat. In addition, the carrot genetic transformation system depending on Agrobacterium has been established [4, 6]. Thus, carrot is a very valuable target for metabolic engineering to improve its nutritional quality and commercial value.

Betalain, which only exists in plants belonging to the order Caryophyllales, is a natural pigment different from carotenoids and flavonoids [7]. It has attracted considerable interest because of its important physiological function and nutritional value. Betalain, which exhibits a huge structural diversity, can be classified into betacyanins (red-violet) and betaxanthins (yellow-orange) according to their molecular structures and light absorption [8]. Betanin (betanidin-5-O–glucoside) is the most well-known betacyanin in the plant kingdom [9]. Currently, the demand for natural food colorant is growing rapidly. Red beet (*Beta vulgaris* L.) is the most main source of betalain for natural pigment extraction. Betanin, making up most of the pigments in red beetroot, has been approved by the U.S. Food and Drug Administration as a food additive [10]. Owing to continuous improvements in the life quality of human, the demand for new alternatives to synthetic colorants in food applications is increasing out of concern for the potential health risks of artificial pigments [11]. However, the natural sources of betalain are limited, thereby hampering its application. Genetic engineering is one of the feasible approaches to develop novel plants as betalain sources.

The discovery of genes and biosynthetic pathways provided the possibility of developing genetically engineered plants for producing betalain. The biosynthesis of betalain in plants has been gradually elucidated. Tyrosine is the direct precursor for betalain biosynthesis and is converted to L-3,4-dihydroxyphenylalanine (L-DOPA). As the common chromophore, betalamic acid is generated from L-DOPA. L-DOPA also can be oxidized to *cyclo*-DOPA, which spontaneously condenses with betalamic acid forming the betacyanin precursor betanidin. The type of distinct ligands conjugated to betalamic acid determines the fate of products, which belong to betacyanin (*cyclo*-DOPA) or betaxanthin (amine or amino acid). The main enzymatic reactions are as follows: (1) tyrosinase or cytochrome P450 enzyme: tyrosine is hydroxylated to L-DOPA; (2) DOPA 4,5-dioxygenase (DOD): betalamic acid is oxidated to L-DOPA; (3) glycosyltransferases: glycosylation modification of *cyclo*-DOPA or betanidin [7, 12].

The genes involved in betalain biosynthetic pathway have been successively cloned and characterized. Tyrosinase is a monooxygenase that widely exist in nature and involved in the biosynthesis of melanin [13]. A tyrosinase gene from *Lentinula edodes* has already been reported to take part in the synthesis of betalain [14]. Certain cytochrome P450 enzymes from Caryophyllales plants exhibit tyrosine hydroxylation activity, and the synthesis of betalain can be blocked by knocking out these genes [15–17]. The DOD gene from *Amanita muscaria* (GenBank: Y12886.1) was the first cloned gene of the betalain pathway [18]. This gene sequence is 1629 bp long including five short introns and encodes for an enzyme consisting of 228 amino acids. The first DOD gene in higher plants (GenBank: AJ580598.1) was cloned from *Portulaca grandiflora* [19]. Subsequently, other DOD genes isolated from *Mirabilis jalapa*, *B. vulgaris*, *Parakeelya mirabiliswere*, and *Anabaena cylindrica*, were cloned and validated [20–23]. Glycosylation is performed by two distinct types of glycosyltransferases in different stages, using *cyclo*-DOPA or betanidin as substrate, respectively. In a heterologous system for betanin production, either of these glucosyltransferases can meet the demand for betanin synthesis [24]. The first betanidin 5-*O*-glucosyltransferase (betanidin 5-GT) gene involved in betalain synthesis in plants was cloned from *Dorotheanthus bellidiformis*, and other genes were subsequently isolated from *B. vulgaris* and *Hylocereus megalanthus* [25–27]. The enzyme activity of UDP-glucose:*cyclo*-DOPA 5-*O*-glucosyltransferase (cDOPA5GT) has been found in *M. jalapa*, and the corresponding cDNA fragments were isolated from *M. jalapa* and *Celosia cristata* [28].

**Fig. 1:**
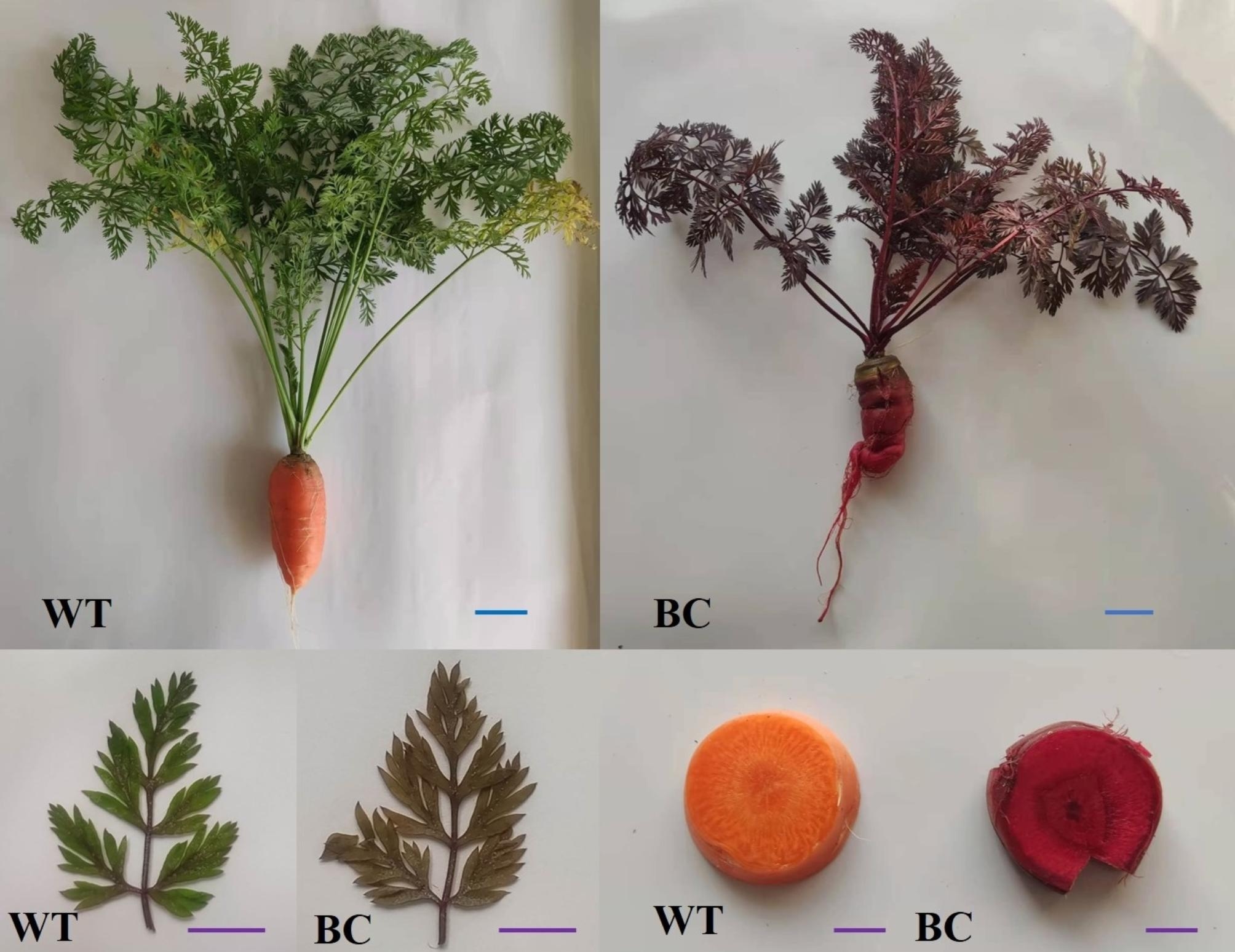
Betanin-transgenic carrots (BC) and non-transgenic carrots (WT) used in this study. Scale bars represent 3 cm (blue) or 1 cm (purple), respectively

Despite that no plant that produces betalains and anthocyanins has been found, betalain can be synthesized in *Arabidopsis thaliana via* expressing a fungal DOD gene and feeding L-DOPA, and subsequent success has been achieved in other plants [29–32]. A novel cytochrome P450 enzyme (CYP76AD1) of beets has dual functions in tyrosine hydroxylation and converting L-DOPA into *cyclo*-DOPA [17]. In our previous study, stably transformed carrot lines containing *CYP76AD1S*, *cDOPA5GTS* and *DODA1S* genes can synthesize and accumulate betanin in the whole plant (Fig. 1). This may improve the nutritional quality of carrot and can be used in extracting betanin from the inedible parts of carrots to obtain further gains. Betalain participates in various physiological activities, including limiting damage caused by wounding and bacterial infiltration, scavenging reactive oxygen species (ROS), responses to salinity stress, and promoting photoprotection [25, 33–35]. As a secondary metabolite, betalain is synthesized from the product of primary metabolism in plants. The accumulation of betanin may affect the metabolism of transgenic carrot lines. Understanding related metabolic pathways facilitates betanin synthesis optimization in transgenic carrots. In addition, genetic transformation manipulation, gene disruption, and genome rearrangements at the insertion site may have unexpected impacts on transgenic plants [36]. As a continuation of our previous work, the antioxidant capacity, antioxidative enzymes activity, and, chlorophyll content of the leaves have been investigated and compared with those of non-transgenic plants. In addition, metabolic changes were also analyzed by transcriptome and metabolomics between betanin-transgenic carrots (BC) and non-transgenic carrots (WT) in this study.

## Results

### Effects of betanin synthesis on chlorophyll accumulation in transgenic carrot

**Fig. 2:**
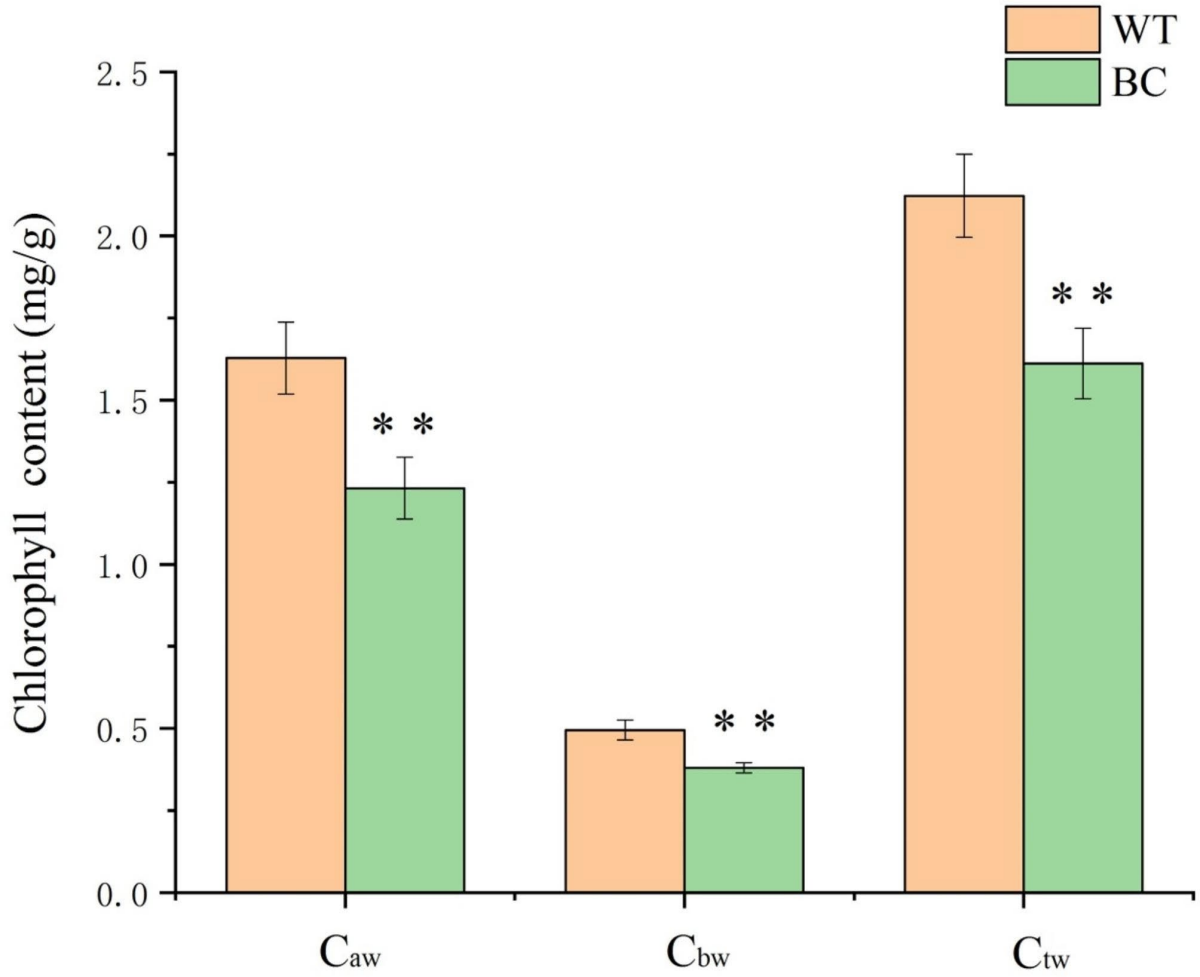
Chlorophyll content of transgenic (BC) and non-transgenic (WT) Leaves. C_aw_: Chlorophyll *a* content, C_bw_: chlorophyll *b* content and C_tw_: chlorophyll *a* + *b* content. Asterisks (*) indicate that the value is significant difference compared to the WT (** *p* < 0.01)

Owing to betanin accumulation, the color of BC transgenic leaves is visibly dark (Fig. 1). The betanin content of transgenic leaves was 11.0 ± 8 µg/g fresh weight. The accumulation of pigments, including chlorophyll and betanin, is the material basis for the formation of transgenic leaf color. We found that the chlorophyll content of transgenic plants was slightly lower than that of WT plants, and the total chlorophyll content was 1.61 and 2.12 mg/g fresh weight, respectively (Fig. 2). This difference and betanin accumulation might have implications for the photosynthetic capacity of transgenic leaves.

### Enhancing total antioxidant capability of transgenic carrot by betanin accumulation

**Fig. 3:**
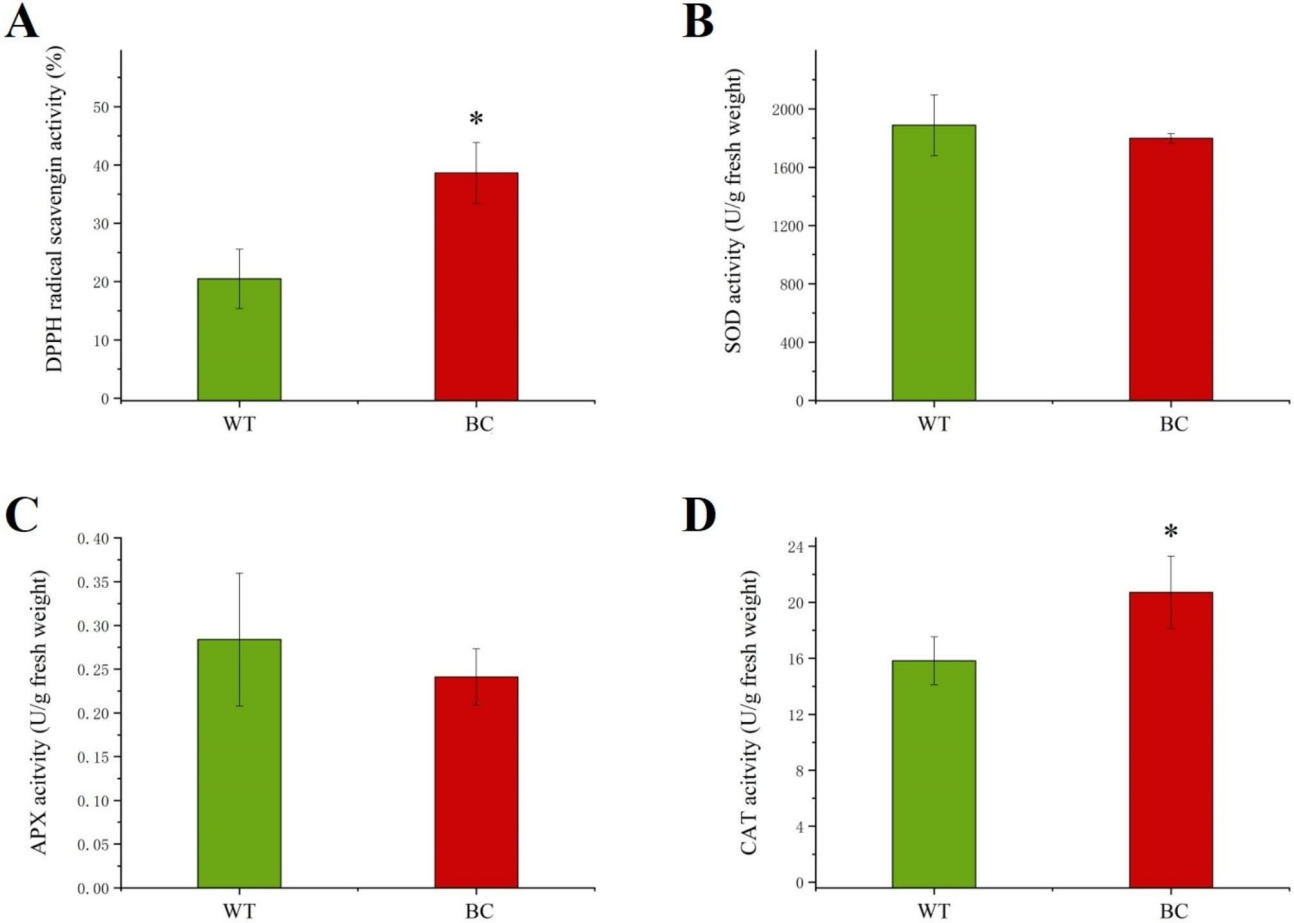
Total antioxidant capability of transgenic (BC) and non-transgenic (WT) Leaves. **A** DPPH radical scavenging ability, **B** superoxide dismutase (SOD) activity, **C** ascorbate peroxidase (APX) activity, and **D** catalase (CAT) activity. Asterisks (*) indicate that the value is significant difference compared to the WT (* *p* < 0.05)

Betanin possessed a strong free radical scavenging ability. The influence of betanin accumulation in carrot leaves was analyzed. The total antioxidant capacity of BC transgenic leaves was tested using the DPPH method. The result established that transgenic leaves possessed a stronger DPPH radical scavenging ability (Fig. 3A).

Antioxidant enzymes, involved in the environmental stress response, are critical components in oxidative stress prevention in plants. Superoxide dismutase (SOD), ascorbate peroxidase (APX), and catalase (CAT) are important antioxidant enzymes. There was no significant difference in SOD and APX activity between BC and WT plants. However, the CAT activity of BC carrots was higher than that of WT carrots, which increased by about 31.2% with significant differences (Fig. 3).

### Global analysis of RNA-seq data and differentially expressed genes (DEGs) in tyrosine metabolism between transgenic and non-transgenic fleshy roots

The tyrosine content of BC and WT groups were 0.78 and 1.81 µg/g dry weight, respectively. Transcriptome profiling was performed to gain insights into molecular changes. Total RNA isolated from BC and WT fleshy roots were sequenced for transcriptomic analysis. A total of 66,666,664 raw reads and 66,455,000 clean reads were obtained, respectively. The Q20 ratio of each sample was 97.99–98.14%, and the Q30 was 94.43–94.83%. GC percentages were relatively consistent (at around 42%) across all samples. By aligning transcript sequences to the reference genome sequences, the rate of total mapping ranged from 88.67 to 89.18%.

**Fig. 4:**
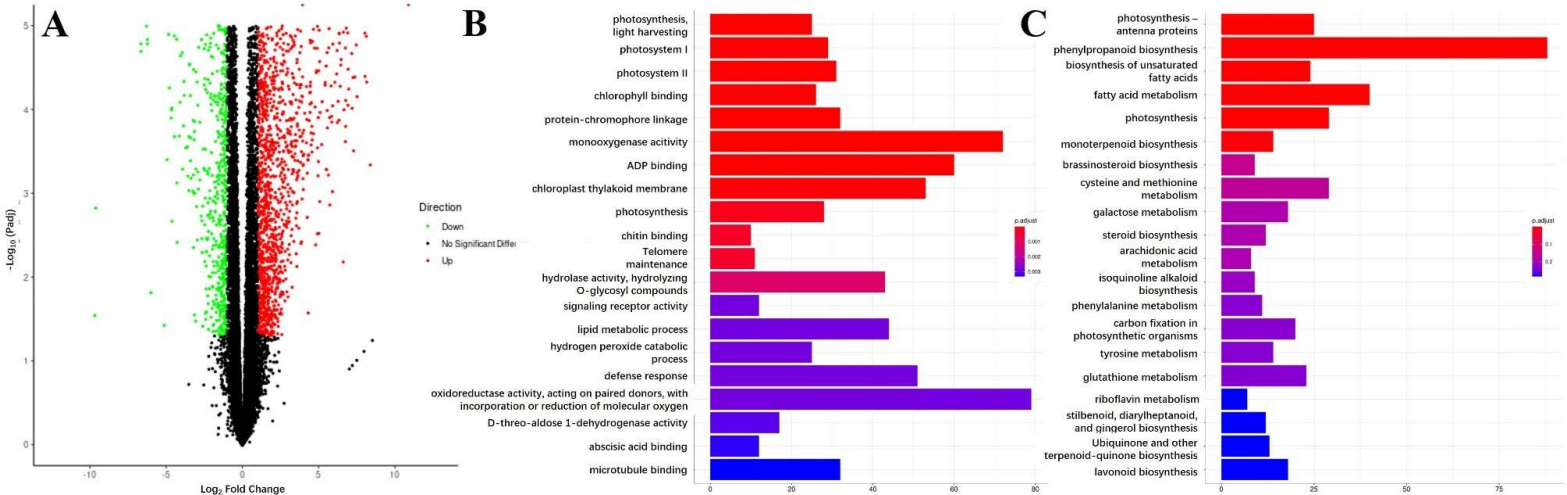
Visualization of transcriptome data information and analytic results (BC vs. WT). **A** Volcano plots showing DEGs, **B** GO enrichment analysis of DEGs, **C** KEGG pathway enrichment analysis of DEGs

A total of 35,073 genes were found, of which 32,113 had functional annotations. To identify alterations in gene expression levels, gene expression was assessed by fragments per kilobase per million bases) (FPKM). Finally, a total of 4,907 DEGs including 3,203 upregulated genes and 1,704 downregulated genes were obtained (Fig. 4A). Gene Ontology (GO) analysis was performed. The top 20 GO terms are presented in Fig. 4B. The DEGs, which were categorized in the ‘oxidoreductase activity, acting on paired donors, with incorporation or reduction of molecular oxygen’, was the largest category. The ‘monooxygenase activity’ was the second group with most genes assigned, followed by ‘ADP binding’, ‘chloroplast thylakoid membrane’, and ‘defense response’. In addition, DEGs in ‘hydrogen peroxide catabolic process’ was also enriched. These may be associated with the antioxidant activity of synthetic betanin, and serve as a significant reflection.

At the same time, we analyzed metabolic pathways based on Kyoto Encyclopedia of Genes and Genomes (KEGG) annotation to obtain significantly enriched pathways. Among the top 20 most enriched pathways, ‘Phenylpropanoid biosynthesis’ was the most representative metabolic pathway, in which a total of 88 DEGs were enriched. ‘Fatty acid metabolism’, ‘Photosynthesis’, ‘Cysteine and methionine metabolism’, and ‘Photosynthesis-antenna proteins’ were the other representative pathways (Fig. 4C).

Fourteen and eight DEGs were enriched in ‘Tyrosine metabolism’ and ‘Phenylalanine/tyrosine/tryptophan biosynthesis’ pathway, respectively. The possible reason for this is that tyrosine is the direct precursor for betanin biosynthesis. The extra consumption of tyrosine and the expression of the exogenous betanin synthesis pathway had significant effects on tyrosine metabolism in BC carrot fleshy roots. Divergent responses in the amino acid metabolisms and secondary metabolite synthesis were observed. Ten unigenes were randomly selected for quantitative real-time PCR (qRT-PCR) to test the RNA-seq data. The result was highly correlated with the RNA-seq data, which confirmed the validity of the RNA-seq data (Figure S1) [see Additional file 1].

### Untargeted metabolome analyses and comparison of tyrosine content between transgenic and non transgenic fleshy roots

**Fig. 5:**
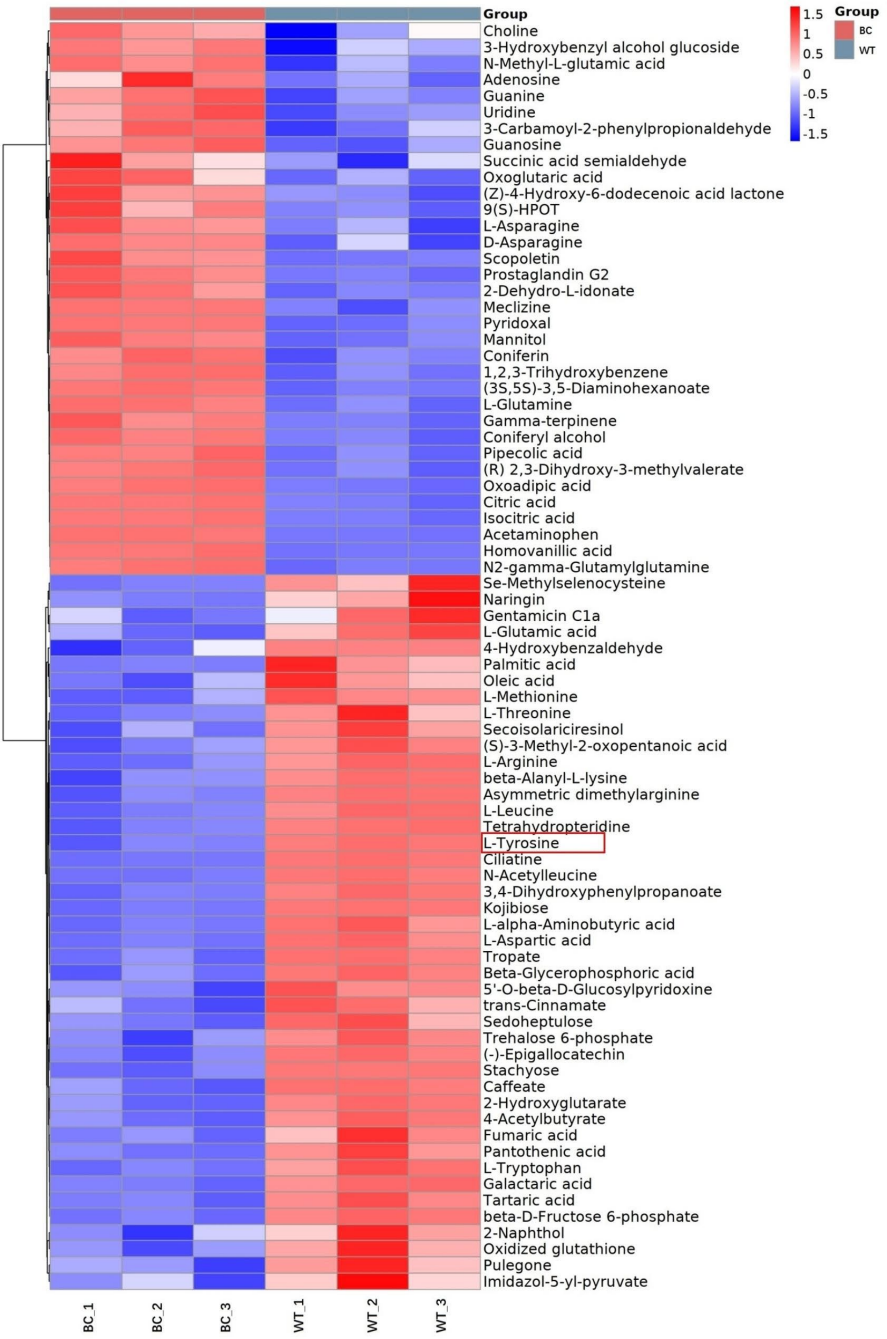
Heat map analysis of all differentially accumulated metabolites

**Fig. 6:**
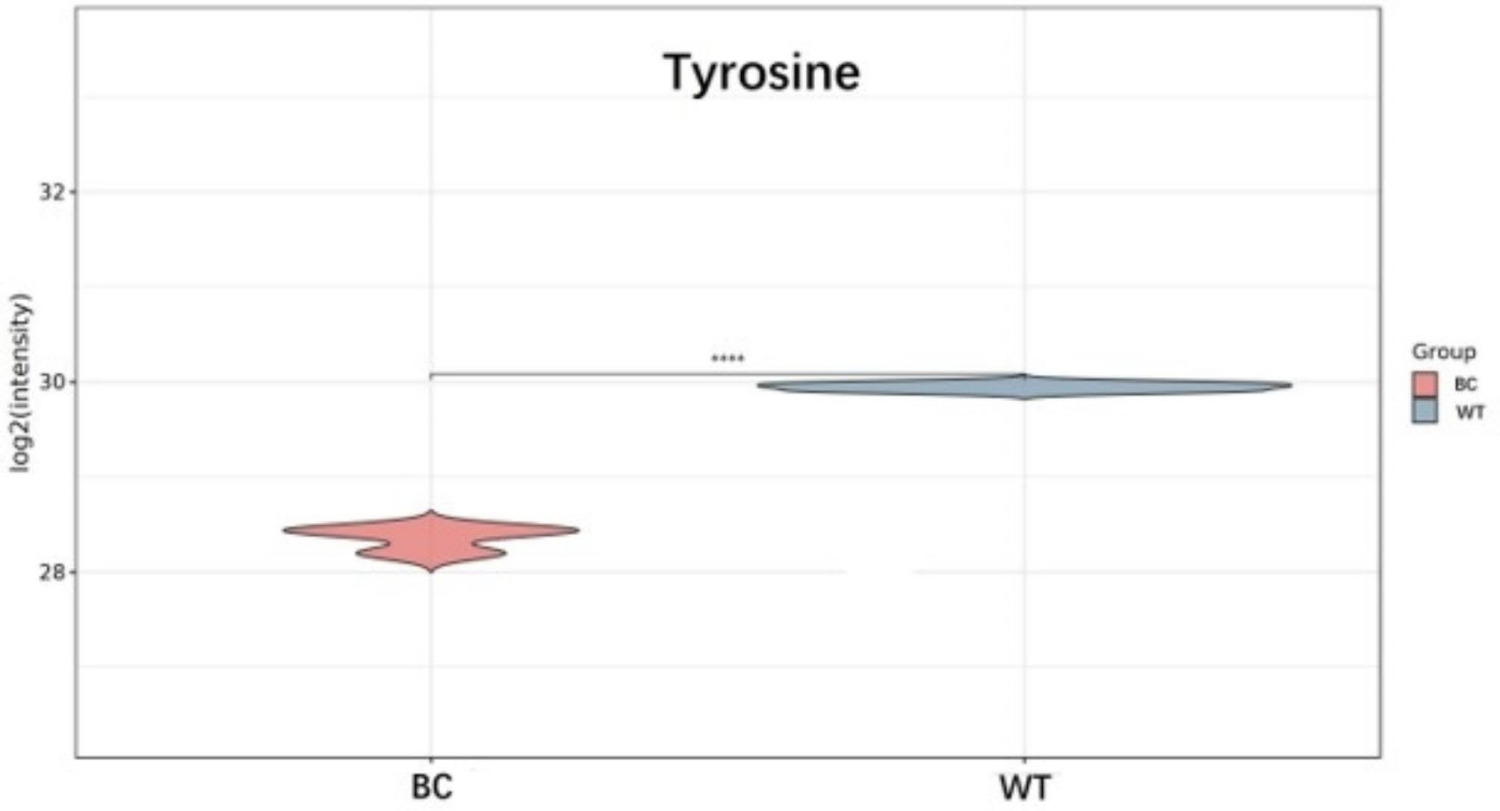
Data represented as violin plots to show distribution of tyrosine data. Asterisks (*) indicate that the value is significant difference compared to the WT (**** *p* < 0.0001)

Metabolomics assessment of carrot samples from BC and WT groups was performed with liquid chromatography coupled with tandem mass spectrometry (LC-MS/MS) in positive and negative ion modes. The metabolites were identified by accuracy mass (< 30 ppm) and MS/MS data which were matched with HMDB (http://www.hmdb.ca), massbank (http://www.massbank.jp/), LipidMaps (http://www.lipidmaps.org), mzclound (https://www.mzcloud.org) and KEGG (http://www.genome.jp/kegg/). All 236 identified metabolites were classified as ‘organooxygen compounds’, ‘fatty acyls’, ‘carboxylic acids and derivatives’, ‘pyridines and derivatives’, ‘benzene and substituted derivatives’, and so on. In total, 78 differentially accumulated metabolites (DAMs) were identified, including 34 DAMs were upregulated and 44 DAMs were downregulated (Fig. 5). Some amino acids, including asparagine, methionine, threonine, arginine, leucine, tyrosine, aspartic acid, and tryptophan, were identified as DAMs. Among them, the content of tyrosine in BC carrot was 41.7% that in WT carrot (Fig. 6).

**Fig. 7:**
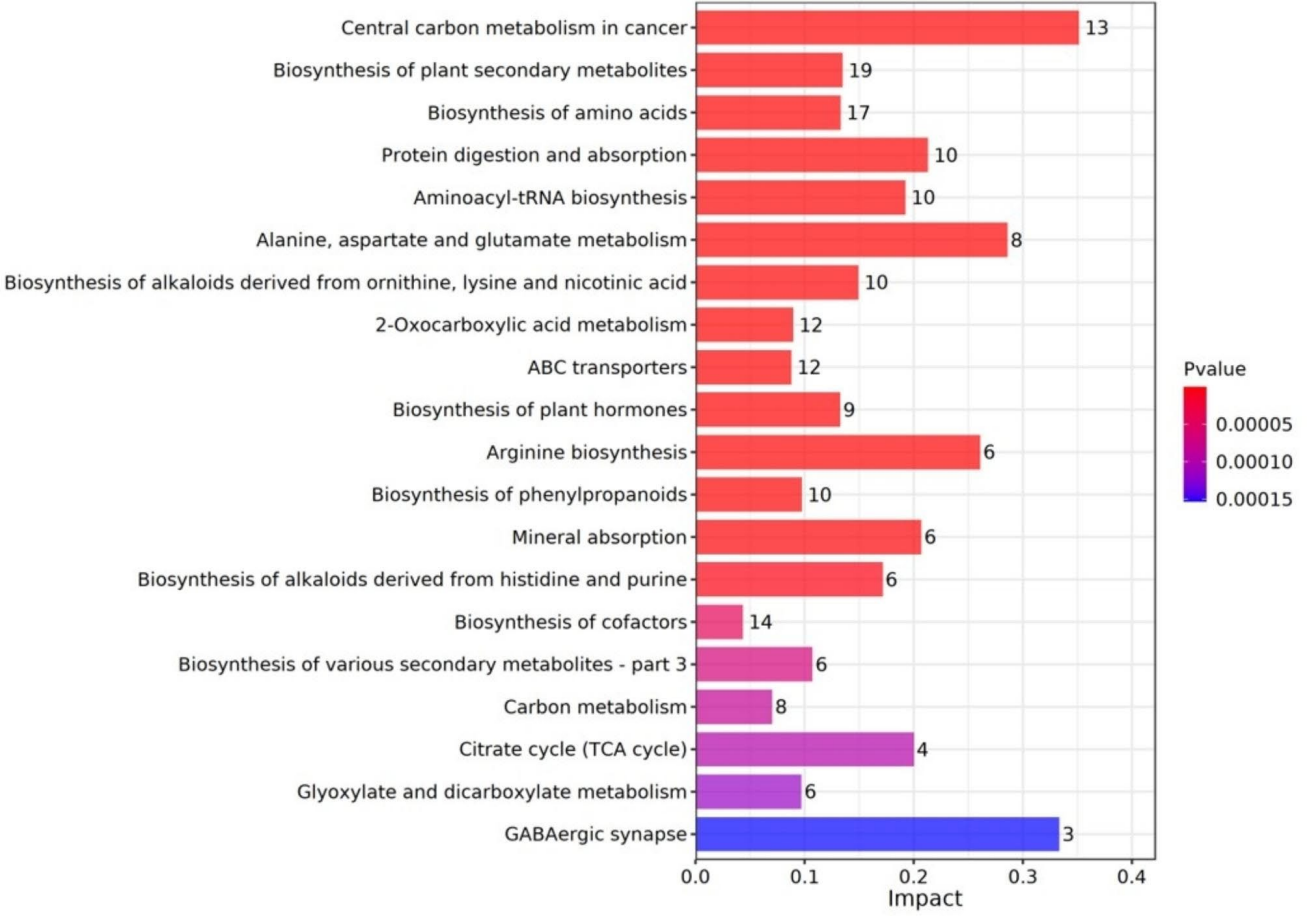
Visualization of metabolomics data information and analytic results (BC vs. WT). Bar graph of the DAMs significantly enriched pathways

Differential metabolites were subjected to pathway analysis by MetaboAnalyst, which combines results from powerful pathway enrichment analysis with the pathway topology analysis. The relative abundance of DAMs was mainly related to ‘central carbon metabolism in cancer’, ‘biosynthesis of plant secondary metabolites’, and ‘biosynthesis of amino acids’ (Fig. 7).

### Conjoint analysis of the transcriptome and metabolome data

**Fig. 8:**
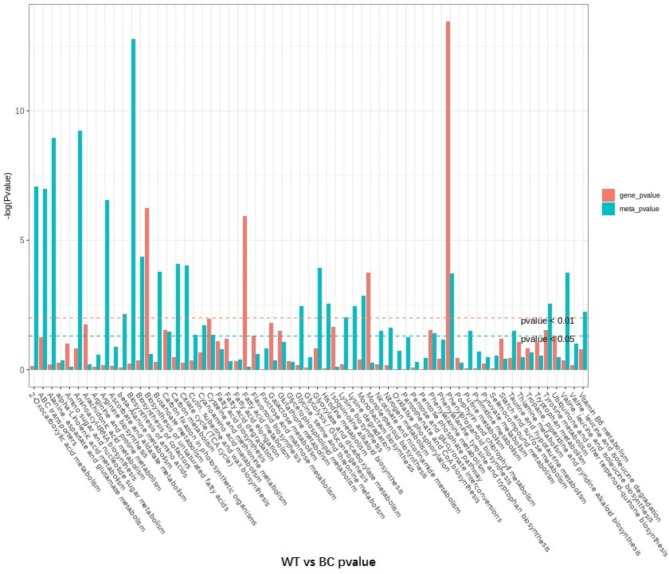
Conjoint KEGG enrichment analysis for DEGs and DAMs

To investigate the relationship of DAMs and DEGs involved in the same KEGG pathway, the co-expression analysis of metabolome and transcriptome was performed using Pearson’s correlation coefficient. ‘tyrosine metabolism’ pathway was enriched by conjoint analysis. In addition, there are other pathways which simultaneously enriched DEGs and DAMs. Only the P-value of ‘phenylpropanoid biosynthesis’ was less than 0.01, and ‘carbon fixation in photosynthetic organisms’, ‘cysteine and methionine metabolism’, and ‘phenylalanine metabolism’ pathways were less than 0.05 (Fig. 8).

## Discussion

Betalain has been demonstrated to have important nutritional value and broad application prospects. The genes of the core pathway of betalain synthesis are now available for genetic engineering toward plants that are unable to synthesize betalain [8, 26, 37, 38]. Betanin synthesis in carrot has been achieved by genetic engineering in our previous study. However, the effects of betanin accumulation and genetic transformation on the metabolism of transgenic carrots are still unclear. Unintended effects, which are not synonymous with harmful or detrimental, widely occur in genetically modified crops [39]. To transgenic plants, it is also necessary to evaluate their potential unintended effects including the impact on metabolic process of transgenic receptors.

Betanin, as food antioxidant additive, is extremely stable during storage at low temperature and alkaline pH [40]. In our preliminary study, the extract of transgenic BC plants showed increased free radical scavenging activity than that of WT plants. Similar results have been reported for the endosperm of genetically modified ‘Betanin Rice’ [41]. Plants typically accumulate ROS under stress conditions and responded against ROS by increasing antioxidant enzyme activities [42]. Only CAT, as an antioxidant enzyme against oxidative stress, shows significant difference between the BC and WT plants. Therefore, we believe that the synthesis of betanin is mainly responsible for the improved antioxidant capacity.

We also found that the color of BC leaves is visibly darker than WT leaves. Leaf color variation was associated with chlorophyll content decrease and betanin accumulation. In addition, DEGs were enriched in ‘photosynthesis-antenna proteins’, ‘photosynthesis’, ‘photosynthesis, light harvesting’, ‘photosystem I’, ‘photosystem II’, ‘carbon fixation in photosynthetic organisms’, and ‘chlorophyll binding’ by data analysis. These results implied that the photosynthesis may be affected by the synthesis and accumulation of betanin in BC plants. Betalain plays a protective role in photosynthesis of *Disphyma austral* under stress condition [35]. Under stress conditions with H_2_O_2_ supplementation, DEGs involved in β-carotene metabolism were upregulated in the unicellular alga *Dunaliella salina*, whereas genes involved in photosynthesis were downregulated [43]. More than 40% of light energy was absorbed by the anthocyanin accumulated in maize leaves [44]. Thus, excess plant pigment such as betanin in leaves may have a negative effect on photosynthesis. Compared to the non-transgenic parental counterpart, the leaf photosynthesis of commercial Bt-transgenic cotton was also affected, and this effect was considered an unintended effect [45]. Our results suggest that the decrease in chlorophyll content and the accumulation of betanin observed in BC plants might have affected their photosynthesis. At the same time, the synthesis of exogenous betanin may also alter plant metabolism. Further studies are therefore needed to better understand the role of betanin biosynthesis in transgenic carrots. To avoid the interference of betanin accumulation on photosynthesis, the site of betanin synthesis should be designed by a tissue-specific promoter to limit this negative effect.

As a basic building block of protein, tyrosine is the direct precursor for the synthesis of betanin. The expression of betalain biosynthetic pathway may affect tyrosine metabolism in BC plants. Transcriptome and metabolomic analyses were performed on the fleshy roots of BC and WT carrots to determine the effects caused by genetic transformation. DEGs were assigned to ‘tyrosine metabolism’ and ‘phenylalanine, tyrosine and tryptophan biosynthesis’. In addition, we also found that 17 DAMs were enriched in ‘biosynthesis of amino acids’. Decreased accumulation of methionine, threonine, arginine, leucine, tyrosine, aspartic acid, and tryptophan was observed in the BC plants. Tyrosine content of BC group was only one half that of WT group by free amino detection. Betanin synthesis should be an additional tyrosine-consuming process that contributes to the decrease in tyrosine content. Amino acids can react with betalamic acid to produce different betaxanthins [46]. This reaction is a possible reason for the lower content of other differential amino acids. This speculation should be validated by further analysis and follow-up experiments. Chorismate, the product of shikimate pathway, is the substrate for branch pathways responsible for the synthesis of phenylalanine/tyrosine and tryptophan. The competition for chorismate may be the cause of the lower content of tryptophan. However, no clear differences in phenylalanine content were found between the WT and BC plants. Amino acids serve to maintain fundamental life processes. These results clearly demonstrate the impact of exogenous betalain pathway on amino acid metabolism, especially tyrosine metabolism.

Omics techniques provide powerful approaches for analyzing unintended effects in genetically engineered plants [47].According to transcriptome and metabolomic analyses, the unintended variations involved in the synthesis of diverse metabolites, including unsaturated fatty acids, monoterpenoid, steroid, flavonoid, amino acids, hormones, and other plant secondary metabolites. The leading cause of unintended variations and effects is the random insertion of exogenous DNA fragments, which alter the expression of intrinsic genes. Differentially expressed transcripts were mainly implicated in the alteration of stress/defense responses and amino acids metabolism of genetically modified rice leaves contain a single site insertion of *cry1Ab* gene [48]. Slice differences were detected in metabolic profiles by *Cry1C* gene transformation, but metabolic activity showed significant response to rice dwarf virus infection [49]. Genetic manipulation and in vitro culture are the other major sources of unintended effects [36, 50]. Integration analysis of the transcriptome and metabolome profiles revealed that ‘phenylpropanoid biosynthesis’ pathway was significantly enriched. Phenylpropanoid metabolism, as one of the most important metabolic process in plants, produces metabolites including lignin, flavonoids, lignans, phenylpropanoid esters, hydroxycinnamic acid amides, and sporopollenin [51]. The significantly changes in phenylpropanoid metabolism might account for the alternations of BC transgenic carrots.

## Conclusions

Comparative analysis showed that betanin synthesis in BC transgenic carrot leaves can significantly improve their antioxidant capacity and affect photosynthesis. Based on the conjoint analysis of the transcriptome and metabolome data, tyrosine metabolism is significantly influenced by the exogenous betalain pathway. In addition, unintended effects involved in the synthesis of diverse metabolites were also found. Changes in phenylpropanoid metabolism were considered an important reason for difference between BC and WT carrots.

## Methods

### Plant materials and growth conditions

The carrot cultivar ‘Sanhongliucun’ was used for genetic transformation experiments. Expression vector construction and carrot genetic transformation were performed according to the previously described method [41, 52]. The transgenic lines, which can synthesize betanin depending on expressing codon optimized *BvDODA1* (GenBank No.: HQ656021.1), *BvCYP76AD1* (GenBank No.: HQ656023.1), and *MjcDOPA5GT* (GenBank No.: AB182643.1), were obtained in our previous works. The optimized genes were renamed *CYP76AD1S*, *cDOPA5GTS* and *DODA1S*, respectively. The sequences of these genes were shown in additional material [see Additional file 1]. Transgenic plants were grown in a controlled artificial climatic chamber under the same conditions as previously described [49]. Carrot cv. ‘Sanhongliucun’ and the transgenic carrots were deposited at the State Key Laboratory of Crop Genetics and Germplasm Enhancement, Nanjing Agricultural University (32° 04′ N, 118° 85 ′E). The leaves and fleshy roots were used to analyze differences between the BC and WT carrots (Fig. 1). Samples were taken from plants (8–12 weeks after transplanting) with consistent growth. Unless otherwise stated, all chemicals were obtained from commercial sources and were of analytical grade.

### Extraction and detection of betanin

For the extraction and detection of betanin, tissues were individually ground with liquid nitrogen, and the homogenate was resuspended in 0.1% formic acid solution. Then, the samples were subjected to LC-MS to detect betanin according to the method described previously [41]. Quantitation was based on standard curves from authenticated standards (Sigma, China).

### Analysis of leaf antioxidant activities and chlorophyll content

The DPPH method was used to assess the total antioxidant capacity of BC and WT leaf tissues [53]. The basal activities of SOD, CAT, and APX were analyzed and compared. Superoxide Dismutase (SOD) assay kit (WST-1 method), Catalase (CAT) assay kit (Visible light) and Ascorbate peroxidase (APX) test kit were purchased from Nanjing Jiancheng Bioengineer Institute. The specific operation procedures refer to the instructions attached to the kit with slight modifications. The chlorophyll content was detected by spectrophotometry. Chlorophyll was isolated with 80% acetone, and chlorophyll concentration was detected with a microplate reader (Infinite M200, Tecan Group Ltd.) [54].

### Transcriptome analysis of the transgenic and non-transgenic fleshy roots

Purified mRNA was extracted from BC and WT fleshy roots and fragmented into small pieces with a fragment buffer. Then, first-strand cDNA was generated by random hexamer-primed reverse transcription, followed by a second-strand cDNA synthesis. The fragments of cDNA obtained from previous steps were amplified by PCR, and validated on the Agilent Technologies 2100 bioanalyzer for quality control. The double-strand PCR products were denatured by heating and circularized by the splint oligo sequence to get the final library, and the single stranded circular DNA was formatted as the final library. After removing adapters and low-quality sequences, the clean reads were mapped to the carrot genome using HISAT. The gene expression levels in stressed samples were compared with those in control samples in order to identify the DEGs. The DEGs were detected based on the parameters: Fold change ≥ 2.00 and Probability ≥ 0.8 with a significant false discovery rate-adjusted P-value (FDR) < 0.05 based on the three biological replicates. Gene Ontology (GO) and Kyoto Encyclopedia of Genes and Genomes (KEGG) enrichment analyses for the DEGs were performed using the cluster Profiler version 3.8 [55–57]. To test the RNA-seq data, 10 genes *DCAR_028466*, *DCAR_006579*, *DCAR_022568*, *DCAR_029938*, *DCAR_006723*, *DCAR_012435*, *DCAR_013894*, *DCAR_028849*, *DCAR_004554*, *and DCAR_019343* were randomly selected for qRT-PCR. *ACTIN* was used as an internal control [58]. qRT-PCR was conducted using TaKaRa SYBR Premix Ex Taq (TaKaRa, Dalian, China) equipped with a MyIQ Real-Time PCR Detection System (Bio-Rad, CA, USA).

### Tyrosine content and metabolomics analysis of the transgenic and non-transgenic fleshy roots

The tyrosine content was quantified by an automatic amino acid analyzer (L-8900, Hitachi, Tokyo, Japan), according to the method described in Cao et al. [59]. For metabolomic analysis, carrot fleshy roots were homogenized into powder in liquid nitrogen and then the metabolites were extracted with methanol/acetonitrile/H_2_O solution (2:2:1, v/v/v). After a series of treatments, the samples were re-dissolved in 100 µL of acetonitrile/water (1:1, v/v) solvent and transferred to LC vials for LC-MS analysis. For the untargeted metabolomics of polar metabolites, extracts were analyzed using a quadrupole time-of-flight mass spectrometer (Sciex TripleTOF 6600) coupled to hydrophilic interaction chromatography *via* electrospray ionization in Wuhan Benagen Technology Co., Ltd. The raw MS data (wiff.scan files) were converted to mzXML files using Proteo Wizard MSConvert before importing into freely available XCMS software. Compound identification of metabolites by MS/MS spectra with an in-house database established with available authentic standards. For KEGG pathway annotation, the metabolites were blasted against the online KEGG database. To further explore the impact of differentially expressed metabolites, enrichment analysis was performed.

### Integrative metabolome and transcriptome analysis

All detected metabolites and genes were subjected to data analysis. Pearson correlation coefficient was calculated using the python package SciPy (version 1.3.1) with correlation coefficients > 0.8 and P < 0.05 as the selection criteria. Joint pathway enrichment analysis was carried out according to metabolite and gene enrichment analysis results. Pathways with enrichment estimates for genes and metabolites were selected and used in plotting a bar graph.

### Electronic supplementary material

Below is the link to the electronic supplementary material.

Supplementary Material 1

## Data Availability

The datasets used and/or analyzed during the current study are available from the corresponding author on reasonable request. The datasets generated and/or analysed during the current study are available in the NCBI Sequence Read Archive (SRA) [PRJNA960884] and Gene Expression Omnibus (GEO) [GSE231349] repositories. Carrot ‘Kurodagosun’ and BC transgenic carrots were deposited at the State Key Laboratory of Crop Genetics & Germplasm Enhancement and Utilization, Nanjing Agricultural University.

## Abbreviations

L-DOPA: L-3,4-dihydroxyphenylalanine
FDA: U.S. Food and Drug Administration
DOD: DOPA 4,5-dioxygenase
cDOPA5GT: UDP-glucose:*cyclo*-DOPA 5-Oglucosyltransferase
BC: Betanin-transgenic carrots
WT: Non-transgenic carrots
SOD: Superoxide dismutase
APX: Ascorbate peroxidase
CAT: Catalase
DEGs: Differentially expressed genes
FPKM: Fragments Per Kilobase per Million bases
DAMs: Differentially accumulated metabolites
ROS: Reactive oxygen species
GO: Gene Ontology
KEGG: Kyoto Encyclopedia of Genes and Genomes
