# Supplementary material for: Effect of betanin synthesis on photosynthesis and tyrosine metabolism in transgenic carrot
Source: BMC Plant Biol. 2023 Aug 24;23:402. doi: 10.1186/s12870-023-04383-9 (PMC10464428; doi:10.1186/s12870-023-04383-9)
Supplement: Supplementary file 1 — Supplementary Material 1 [file 12870_2023_4383_MOESM1_ESM.docx]

**Supporting Information**

1. **Sequences of genes *CYP76AD1S***

1 ATGGATCATG CAACTTTGGC AATGATCCTT GCAATCTGGT TCATCTCTTT

51 TCACTTCATC AAACTGCTGT TCTCCCAACA GACTACCAAA CTGCTTCCAC

101 CTGGTCCAAA GCCATTGCCA ATCATCGGTA ACATCTTGGA AGTCACCACC

151 ACCACCACTG ATGATGTCCT TGATGTTCTT CTTCAGCTGT TCAAGCAGAA

201 CGAACTGACT ATGGGTTCTG TCACCACCAT TGTTGTCTCC TCTGCTGATG

251 TCGCTAAAGA GATGTTCTTG AAGAAGGATC ATCCACTGTC CAACCGTACC

301 ATTCCAAACT CTGTTACTGC TGGTGATCAT CACAAACTGA CTATGTCTTG

351 GTTGCCTGTC TCTCCTAAGT GGCGTAACTT CCGTAAGATC ACTGCTGTCC

401 ACTTGCTGTC TCCTCAACGT CTTGATGCTT GCCAAACCTT CCGTCATGCT

451 AAGGTCCAAC AACTGTACGA GTACGTCCAA GAGTGTGCAC AGAAAGGTCA

501 AGCTGTTGAC ATCGGTAAAG CTGCATTCAC TACTTCTCTG AATCTGTTGT

551 CCAAACTGTT CTTCTCCGTC GAACTGGCAC ACCACAAGTC TCATACTTCT

601 CAAGAGTTCA AGGAACTGAT CTGGAACATC ATGGAAGACA TTGGTAAGCC

651 TAACTACGCT GACTATCTTC CAATCTTGGG TTGTGTTGAT CCATCTGGTA

701 TTCGTCGTCG TTTGGCATGT TCCTTCGATA AGCTGATCGC TGTCTTCCAG

751 GGTATCATCT GTGAACGTCT TGCACCTGAC TCTTCCACCA CCGGTAAGAA

801 GCCTCATCGT TCCTTCGCTA ACCTGGCTAA GATTCATGGT CCATTGATCT

851 CCTTGCGTCT TGGTGAGATC AACCATCTGC TTGTTGACAT CTTCGATGCT

901 GGTACTGACA CCACTTCTTC CACCTTCGAA TGGGTCATGA CCGAGTTGAT

951 CCGTAACCCT GAGATGATGG AGAAGGCACA AGAAGAGATC AAGCAAGTCT

1001 TGGGTAAGGA CAAACAGATC CAGGAGTCTG ACATCATCAA CCTGCCATAC

1051 TTGCAAGCTA TCATCAAAGA AACCCTGCGT CTTCATCCAC CAACTGTCTT

1101 CCTGTTGCCA CGTAAAGCTG ACACTGATGT TGAACTGTAC GGTTACATTG

1151 TCCCTAAAGA TGCACAGATC CTGGTCAACT TGTGGGCAAT CGGTCGTGAT

1201 CCTAACGCAT GGCAGAACGC TGACATCTTC TCTCCTGAAC GTTTCATCGG

1251 TTGTGAGATC GATGTCAAAG GTCGTGACTT CGGTCTGTTG CCATTCGGTG

1301 CTGGTCGTCG TATCTGTCCT GGTATGAATC TGGCTATTCG TATGTTGACC

1351 TTGATGCTGG CTACCTTGCT TCAGTTCTTC AACTGGAAGC TGGAAGGTGA

1401 CATCTCTCCA AAGGACTTGG ACATGGATGA GAAGTTCGGT ATCGCATTGC

1451 AGAAGACCAA GCCATTGAAA CTGATTCCAA TCCCACGTTA CTAA

1. **Sequences of genes *cDOPA5GTS***

1 GGATCCATGA CTGCAATCAA GATGAACACT AACGGTGAAG GTGAGACTCA

51 ACATATCCTG ATGATCCCAT TCATGGCACA AGGTCACTTG CGTCCATTCC

101 TTGAGCTTGC AATGTTCCTG TACAAGCGTT CACATGTGAT CATCACTCTG

151 CTGACTACTC CACTGAACGC TGGTTTCCTT CGTCATCTGC TGCATCATCA

201 TTCATACTCA TCATCTGGTA TCCGTATCGT GGAGTTGCCA TTCAACTCAA

251 CTAATCATGG TCTGCCACCT GGTATCGAGA ACACTGACAA GCTGACTCTG

301 CCACTTGTGG TGTCACTGTT CCATTCAACT ATCTCACTTG ACCCACATCT

351 TCGTGACTAC ATCTCACGTC ATTTCTCACC TGCACGTCCA CCACTGTGTG

401 TGATCCATGA TGTGTTCCTT GGTTGGGTTG ATCAGGTGGC TAAGGATGTT

451 GGTTCAACTG GTGTTGTGTT CACTACTGGT GGTGCATACG GTACTTCTGC

501 ATACGTGTCT ATCTGGAACG ATCTGCCACA TCAGAACTAC TCTGATGACC

551 AAGAGTTCCC ACTTCCTGGT TTCCCTGAGA ACCACAAGTT CCGTCGTTCA

601 CAACTGCATC GTTTCCTGCG TTATGCTGAT GGTTCTGATG ACTGGTCAAA

651 GTACTTCCAA CCACAACTGC GTCAGTCAAT GAAGTCATTC GGTTGGCTGT

701 GCAACTCTGT TGAGGAGATC GAGACTCTTG GTTTCTCAAT CCTTCGTAAC

751 TACACTAAGC TGCCTATCTG GGGTATCGGT CCACTGATCG CATCACCTGT

801 TCAACATTCA TCATCTGACA ACAACTCAAC TGGTGCTGAG TTCGTTCAGT

851 GGTTGTCACT GAAGGAGCCT GACTCTGTGT TGTACATCTC ATTCGGTTCA

901 CAGAACACTA TCTCACCAAC TCAGATGATG GAACTTGCTG CTGGTCTGGA

951 GTCATCTGAG AAGCCATTCT TGTGGGTGAT CCGTGCACCA TTCGGTTTCG

1001 ACATCAACGA GGAGATGCGT CCTGAGTGGC TTCCTGAGGG TTTCGAGGAG

1051 CGTATGAAGG TGAAGAAGCA AGGTAAGCTG GTGTACAAGC TGGGTCCACA

1101 GTTGGAGATC CTGAACCATG AGTCAATCGG TGGTTTCTTG ACTCATTGCG

1151 GTTGGAACTC AATCCTTGAG TCACTTCGTG AAGGTGTTCC TATGTTGGGT

1201 TGGCCACTGG CTGCTGAACA AGCATACAAC CTGAAGTACT TGGAGGATGA

1251 GATGGGTGTT GCTGTTGAGT TGGCACGTGG TCTGGAAGGT GAAATCTCAA

1301 AGGAGAAGGT GAAGCGTATT GTGGAGATGA TCTTGGAGCG TAATGAAGGT

1351 TCAAAGGGTT GGGAGATGAA GAACCGTGCT GTTGAGATGG GTAAGAAGCT

1401 GAAGGACGCT GTGAACGAGG AGAAGGAACT GAAGGGTTCA TCTGTGAAGG

1451 CAATCGACGA CTTCTTGGAT GCTGTGATGC AAGCTAAGCT GGAACCATCA

1501 CTTCAGTAAG AGCTC

1. **Sequences of genes *DODA1S***

1 ATGAAGATGA TGAATGGTGA AGATGCAACT GATCAGATGA TCAAAGAATC

51 CTTCTTCATC ACTCATGGTA ATCCAATCTT GACTGTTGAA GACACCCATC

101 CATTGCGTCC ATTCTTCGAG ACTTGGCGTG AGAAAATCTT CTCTAAGAAG

151 CCTAAGGCAA TCCTGATCAT CTCTGGTCAT TGGGAGACTG TCAAACCAAC

201 TGTCAATGCT GTCCATATCA ACGACACTAT CCATGACTTC GATGACTACC

251 CTGCTGCTAT GTACCTGTTC AAGTACCCTG CACCTGGTGC ACCAGAACTG

301 GCACGTAAAG TCGAGGAGAT TCTGAAGAAG TCTGGTTTCG AGACTGCTGA

351 GACTGATGAA AAGCGTGGTC TGGATCATGG TGCATGGGTT CCACTGATGC

401 TGATGTATCC TGAGGCTGAC ATCCCTGTCT GCCAGCTGTC TGTTCAACCA

451 CATCTGGATG GTACTTACCA CTACAACTTG GGTCGTGCAC TGGCACCATT

501 GAAGAATGAT GGTGTCTTGA TCATCGGTTC TGGTTCTGCA ACTCATCCAC

551 TGGATGAGAC TCCACACTAC TTCGACGGTG TTGCACCTTG GGCTGCTGCA

601 TTCGACTCTT GGCTTCGTAA AGCACTGATC AACGGTCGTT TCGAAGAAGT

651 CAACATCTAC GAGACCAAAG CACCAAACTG GAAACTGGCA CATCCATTCC

701 CTGAACACTT CTATCCACTG CATGTTGTTC TTGGTGCTGC TGGTGAGAAG

751 TGGAAGGCTG AGCTGATTCA TTCTTCTTGG GATCATGGTA CTCTGTGTCA

801 TGGTTCCTAC AAGTTCACCT CTGCTTAA

Table S1 Primer sequences for fluorescence quantitative PCR

| **Gene** | **Sequences of the primers** |
| --- | --- |
| *DCAR_028466* | F  5' AGAACTCGACATCAGGTGCAA 3' |
|  | R  5' AGGCTGGTCCGAGGTACAAT 3' |
| *DCAR_006579* | F 5' CGGAGGAGCATGTTGTGAGT 3' |
|  | R 5' TTGCAGGCAAAAACAGTCGAG 3' |
| *DCAR_022568* | F 5' CAGAGGAGGGGAGCCTCATT 3' |
|  | R 5' GGAACTGCCCACATTCTGTC 3' |
| *DCAR_029938* | F 5' ACTTCCTGGTTTCCCTGCTC 3' |
|  | R 5' AGCCAAAGCACAAGAGGGTC 3' |
| *DCAR_006723* | F 5' AACCCCAGACCATTGGAAGC 3' |
|  | R 5' AGCTCCATTACCCTCCCACT 3' |
| *DCAR_012435* | F 5' GAGATAGGCTGGCCCACAAA 3' |
|  | R 5' ACACTAACACTGGCACTGGG 3' |
| *DCAR_013894* | F 5' TGACGACGGTGCTCTTGTTT 3' |
|  | R 5' CAGGAACCTCATCCTCGTCG 3' |
| *DCAR_028849* | F 5' ATCAAACAGGCCGTGACGAT 3' |
|  | R 5' GATGGCTTCGAGGATCTGGG 3' |
| *DCAR_004554* | F 5' ACAAGTGAACAACCCGGCTA 3' |
|  | R 5' TCGTTCAGCAGCAGTAACCT 3' |
| *DCAR_019343* | F 5' GAGCTTGGGAGACAACCGAA 3' |
|  | R 5' TGAACCCAAAGCCAAAACAGA 3 ' |
| *ACTIN* | F 5' CGGTATTGTGTTGGACTCTGGTGAT 3' |
|  | R 5' CAGCAAGGTCAAGACGGAGTATGG 3' |


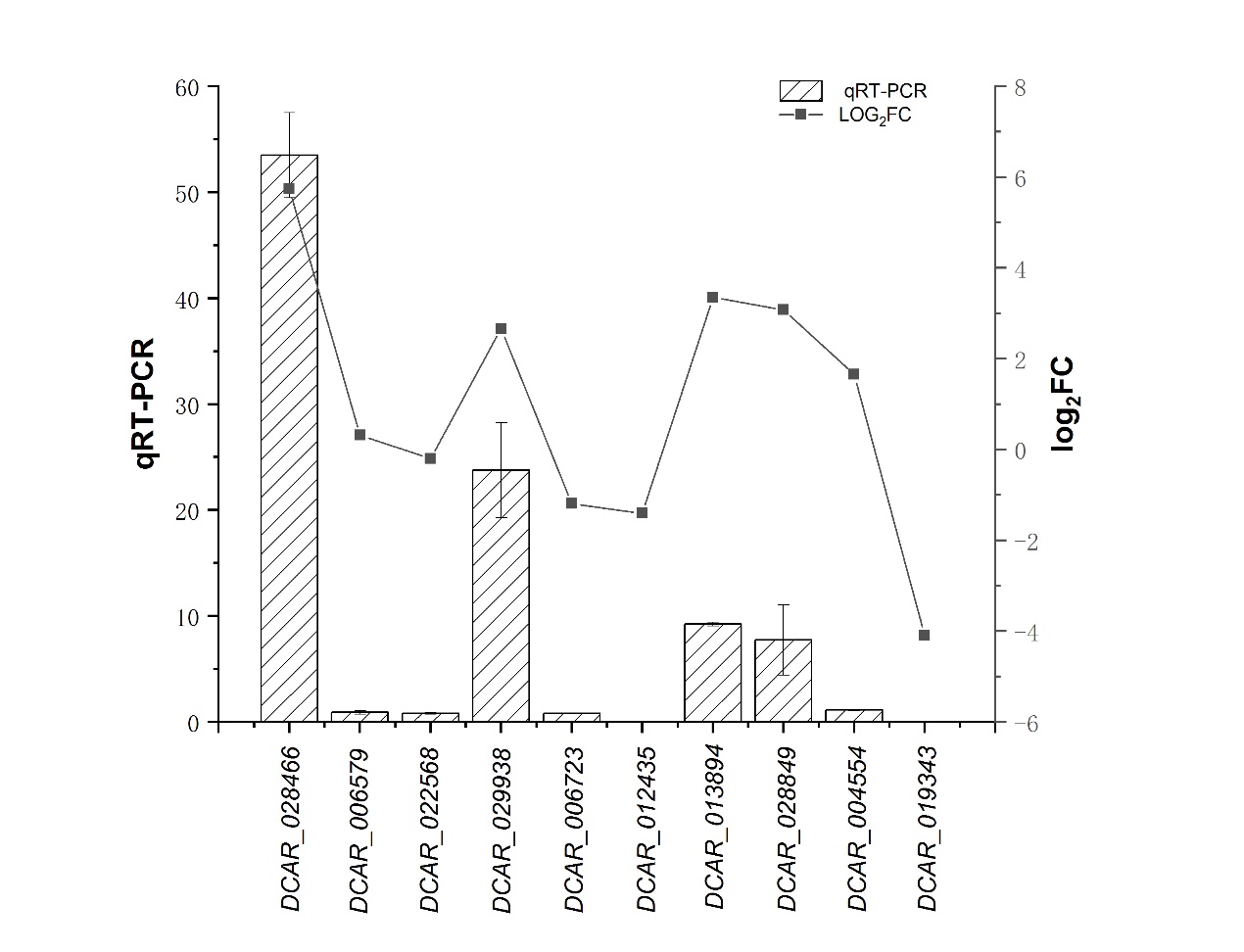


**Figure S1** Validation of gene expression by qRT-PCR analysis. The value of data is the average expression value of the transcriptome and qRT-PCR data. The error

bar represents the standard deviation.
